# A cross-sectional study of ERG expression and the relationship with clinicopathological features of Prostate cancer in Southwestern Uganda

**DOI:** 10.1186/s13000-024-01494-1

**Published:** 2024-05-10

**Authors:** Yekosani Mitala, Brian Ssenkumba, Abraham Birungi, Ritah Kiconco, Marvin Mwesigwa Mutakooha, Raymond Atwine

**Affiliations:** 1 Department of Pathology, Faculty of Medicine, Mbarara University of Science and Technology, Mbarara City, Uganda; 2 Department of Medical Laboratory Science, Faculty of Medicine, Mbarara University of Science and Technology, Mbarara City, Uganda; 3 Department of Biochemistry, School of Health Sciences, Soroti University, Soroti, Uganda; 4 Department of Surgery, Faculty of Medicine, Mbarara University of Science and Technology, Mbarara City, Uganda

**Keywords:** ERG, Immunohistochemistry, Prostate cancer, Uganda

## Abstract

**Background:**

Prostate cancer is the leading cause of cancer-related death and the second most commonly diagnosed cancer among men in Uganda and most countries in Sub-Saharan Africa (SSA). The TMPRSS2-ERG fusion gene is the most common genetic alteration seen among prostate cancer patients. There are several contradicting reports about the association of ERG protein with poor prognosis, high PSA, and Gleason score. This study determined the prevalence of ERG expression and the relationship with PSA, Gleason score, and Age of prostate cancer patients in Southwestern Uganda.

**Methods:**

We reviewed 130 archived prostate biopsy (needle and TURP) specimens from patients of age ≥ 50 years who had a histological diagnosis of prostate cancer. We obtained their biodata, and preoperative PSA, from the archived records. We did Immunohistochemistry (IHC) to determine the prevalence of ERG expression.

**Results:**

The mean patient age in our study was 74.64 ± 10.19 years. Pre-operative PSA levels had been done for 79.2% of the participants. Most cancers (58.46%) were of high grade (grade group 3–5). ERG expression prevalence was 75.4% and its expression was independent of age, re-operative PSA, and Gleason score.

**Conclusion:**

There is a significantly higher prevalence of ERG expression in our study compared to what is reported in other African-based studies. The expression of the ERG is independent of age, Gleason score, and serum PSA levels. A high proportion of our prostate cancer has high-grade disease at the time of diagnosis.

## Introduction

Prostate cancer is the leading cause of cancer-related death among men in Uganda with a very poor prognosis compared to the developed countries [1]. Prior reports attribute such dismal outcomes to late diagnosis, high levels of androgens among Africans, and the lack of screening in the form of PSA testing [2]. Still, they fail to account for the contribution of genetic alterations to the poor outcome. Prostate-specific Antigen (PSA), is used to screen, diagnose, and follow up of prostate cancer patients [3]. The major downside to its use is low specificity leading to overdiagnosis and a lot of negative biopsies [4] and this has led to the introduction of several novel molecular biomarkers to aid prostate cancer management.

Among the new biomarkers is the TMPRSS2-ERG fusion gene, the most common (90% of all genes that fuse with ERG) [5] genetic alteration seen in prostate cancer [6]. TMPRSS2 is a prostate-specific and androgen-response gene that encodes a protein belonging to the serine protease family, which functions in prostate carcinogenesis. The protein functions in conjunction with ETS transcription factors like ERG. ERG on the other hand plays a key regulatory role in cell proliferation, angiogenesis, differentiation and apoptosis, and inflammation. The fusion of these two genes leads to androgen-dependent overexpression of ERG whose transcript has been established as a surrogate marker for the presence of the gene fusion [7]. Fusion occurs either by interstitial deletion (60%) or by translocation (40%) [8]. The deletion type is more common among Africans and in metastatic or castration-resistant prostate cancer leading to an assumption that the deleted segment may have some tumor-suppressive role [9]. The fusion occurs early during the process of carcinogenesis and it can be detected in both high-grade prostatic intraepithelial neoplasms (HGPIN) and overtly invasive adenocarcinomas [10]. Overexpression of ERG may also follow fusion with other genes including SLC45A3, FOXP1, and HERV-K, among others however, this is seen in only about 10% of cases of prostate cancer with ERG overexpression [11].

The prevalence of ERG expression varies globally, ranging from 8 to 83% among some ethnicities [12]. In Sub-Saharan Africa (SSA), the prevalence, and the distribution of this gene are poorly understood. Recent South African and Ghanaian studies observed a prevalence of 13% and 18% respectively [12, 13]. Nothing is known about this gene among East Africans and other regions of SSA. It is suggested that expression of ERG is associated with aggressive tumor behavior, high PSA, and high Gleason score [14, 15]. In a study done by Blackburn et al., (2019), the fusion gene was also associated with younger ages under 65 years. Contrary to the above, the majority of the studies have shown no relationship between the fusion gene, Gleason score, PSA, and age [16–18]. To date, there is no information about the prevalence of ERG expression among prostate cancer patients in Uganda despite the high mortality from the disease. Therefore, this study investigated the prevalence of the ERG expression and its relationship with preoperative PSA, Gleason score, and age of patients with prostate cancer in Southwestern Uganda.

## Methods

### Clinicopathological evaluation

This was a retrospective study conducted on 130 prostate biopsies (needle biopsies and TURP specimens) that already had a diagnosis of prostate cancer at Mbarara University histopathology laboratory “between” March 2016 to March 2020. The sample size was calculated using a formula for a single population proportion with correction for the finite population [19]. The cases were consecutively recruited, excluding exhausted blocks, cases that wouldn’t go past antigen retrieval, and specimens missing source patient age. Clinical information including source patient age, and pre-operative PSA serum measurements were retrieved from the histology request forms. Tissue blocks were retrieved and recut for repeat H&E and IHC staining. Repeat H&E were done for all the blocks following the routine standard operating procedure. We reviewed and confirmed the histology diagnosis, Gleason scores, and grade groups according to the 2014 ISUP grade group system. 10% (10%) of randomly selected H&E slides were sent out to an independent professor of Pathology as a check for quality and consistency of results.

### Immunohistochemistry (IHC)

IHC was done manually with a rabbit monoclonal anti-ERG antibody from Abcam (EPR3864; dilution 1:100). After deparaffinization and hydration of the tissue sections, antigen retrieval was done by heating in an immunoDNA retriever citrate at a PH of 6.1 then cooled for 15 min. Tissues were rinsed 3 times in distilled water, and using a pap pen, circles were drawn around the tissue sections to contain the fluid. Slides were then placed in Tris-buffered saline solution and then rinsed well in 3 jars. Moist paper towels were laid flat on the plastic slide tray and the tissue slides were placed on top of the towels. Peroxidase block was added for 10 min to all slides followed by careful rinsing in Tris buffered saline solution for 3 min. Excess buffer was carefully shaken off the tissue and placed back on the tray. 1–2 drops of the primary antibody (rabbit monoclonal anti-ERG (EPR 3864; dilution 1:100)) were added to the tissue sections. The slide trays were closed and tissues were incubated for 60 min at room temperature followed by careful rinsing in Tris-buffered saline solution 3 times. The secondary antibody (horse-radish peroxidase anti-rabbit) was then added to the sections incubated for 30 min and then rinsed as above. DAB chromogen was then added to the tissue and again incubated for 10 min. DAB waste was collected in a hazardous container. Slides were then rinsed as before. Counterstaining was done with hematoxylin for 1 min, followed by dehydration, clearing, and then coverslipped.

### Interpretation of immunohistochemistry

**Fig. 1:**
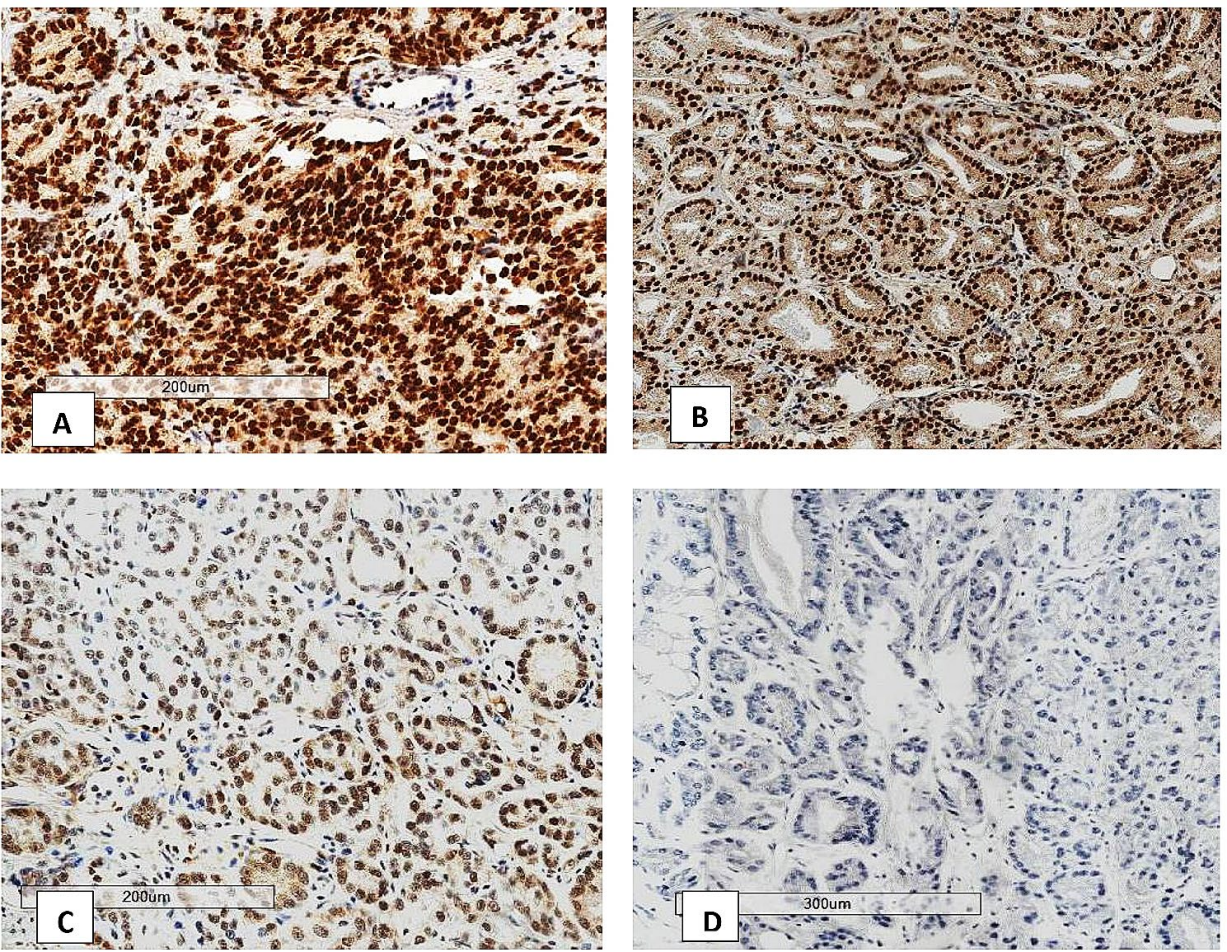
Shows different ERG staining intensities. **A:** Strong ERG staining, **B:** Moderate ERG staining, **C:** Weak ERG staining and **D:** Negative staining

The ERG protein was considered present (Positive) if there is strong or moderate golden-brown staining of the tumor cell nuclei. Weak staining of more than 5% of the tumor cell nuclei was also considered positive. Negative staining was considered if there was no golden-brown staining in the tumor cell nucleus or if there was < 5% staining of target cells. Vascular endothelial cells were used as the internal positive controls that were assigned a staining score of “strongly positive,” and the staining in lymphocytes was assigned a staining score of “weakly positive” (see Fig. 1). An intermediate signal border between a strong and a weak staining signal was assigned as moderate staining. For sections with heterogeneous staining, the highest intensity was considered. Benign glands adjacent to cancerous glands served as internal negative controls.

### Statistical analysis

Variables were analyzed using STATA software version 15.0. Continuous normally distributed variables like age were described using the mean, and categorical variables with proportions and frequencies. The prevalence of ERG expression was calculated as a proportion with its corresponding 95% confidence interval (CI). Bivariable and multivariable logistic regression models were used to determine the relationship between ERG expression and the dependent variables (Age categories, prostate cancer grade groups, and preoperative serum PSA levels). A 5% margin of error was allowed.

## Results

### Source patient characteristics

**Fig. 2:**
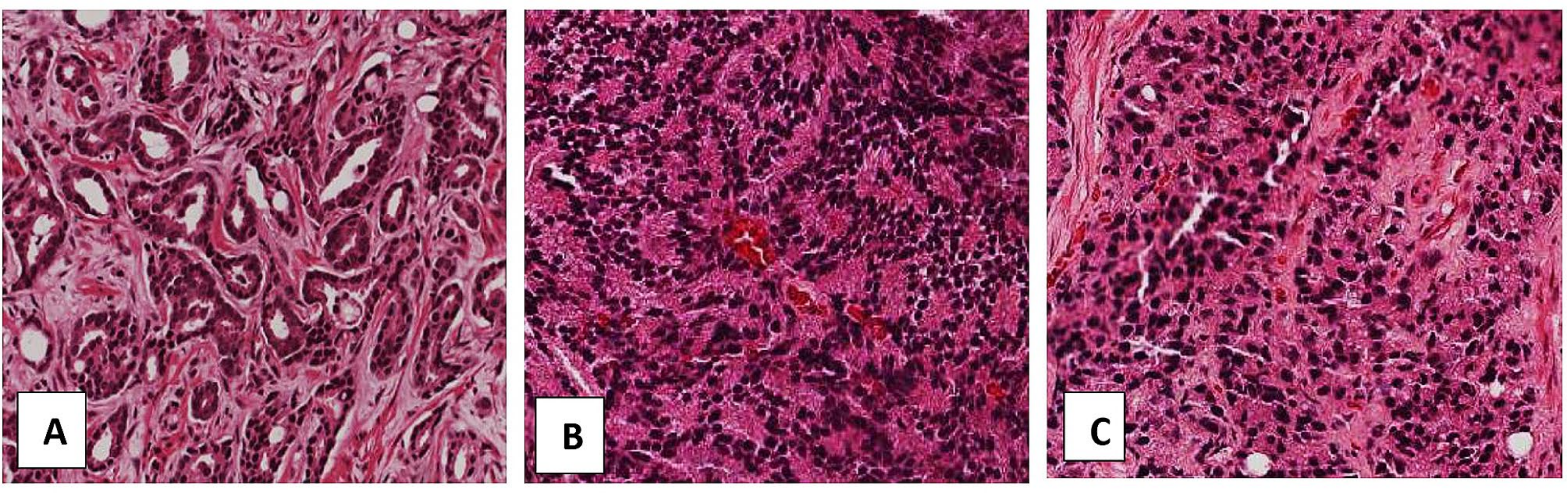
H&E stained sections showing prostate cancer of different Gleason Scores. **A:** Gleason score 3, **B:** Gleason score 4, **C:** Gleason score 5

Our patients’ age ranged between 50 and 106 years, with a mean age of 74.64 years, SD = 10.19. The mean number of cores per slide was 4.13 cores, SD = 1.96. Preoperative PSA was done for 103 of 130 (79.2%) patients with 91.3% of them having preoperative PSA levels over 19ng/ml. Gleason scores (see Fig. 2) were categorized into grade groups based on the 2014 by the International Society of Urologic Pathology (ISUP) classification of prostate cancers. Most prostatic carcinomas are in grade group 5 (37.7%), and grade group 1 (24.6%). High-grade prostate cancer (grade group 3–5) constituted 58.46% of the studied population (see Table 1). Details of pathological and clinical characteristics are summarized in Table 1.

**Table 1:** Pathological and clinical characteristics of biopsies and source patients included in the study

Variable		Total *N* = 130
Age mean (SD)		74.64 (10.19)
Number of cores mean (SD)		4.13 (1.96)
Total PSA (ng/ml)		**103 (79.2%)**
	0–9.0	3 (2.31%)
	9.1–19.0	6 (4.62%)
	> 19	94 (72.31%)
	Missing	**27 (20.77)**
Gleason score Grade groups		
3 + 3	1	32 (24.6%)
3 + 4	2	22 (16.9%)
4 + 3	3	18 (13.8%)
4 + 4, 5 + 3, 3 + 5	4	22 (16.9)
4 + 5, 5 + 4, 5 + 5	5	36 (27.7%)

### Prevalence of ERG expression and the relationship with PSA, gleason score, and age

**Fig. 3:**
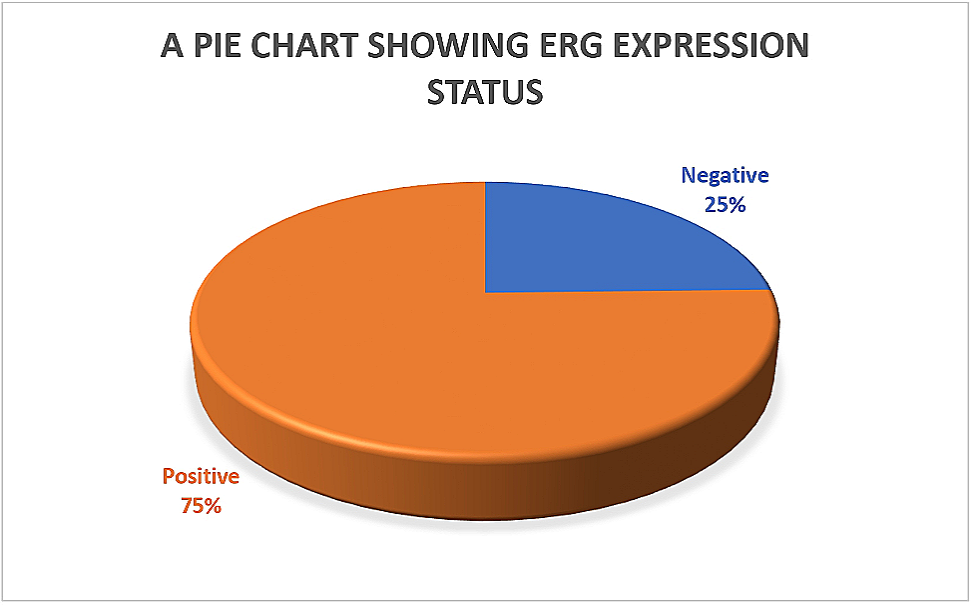
Pie chart showing percentage expression of ERG

**Fig. 4:**
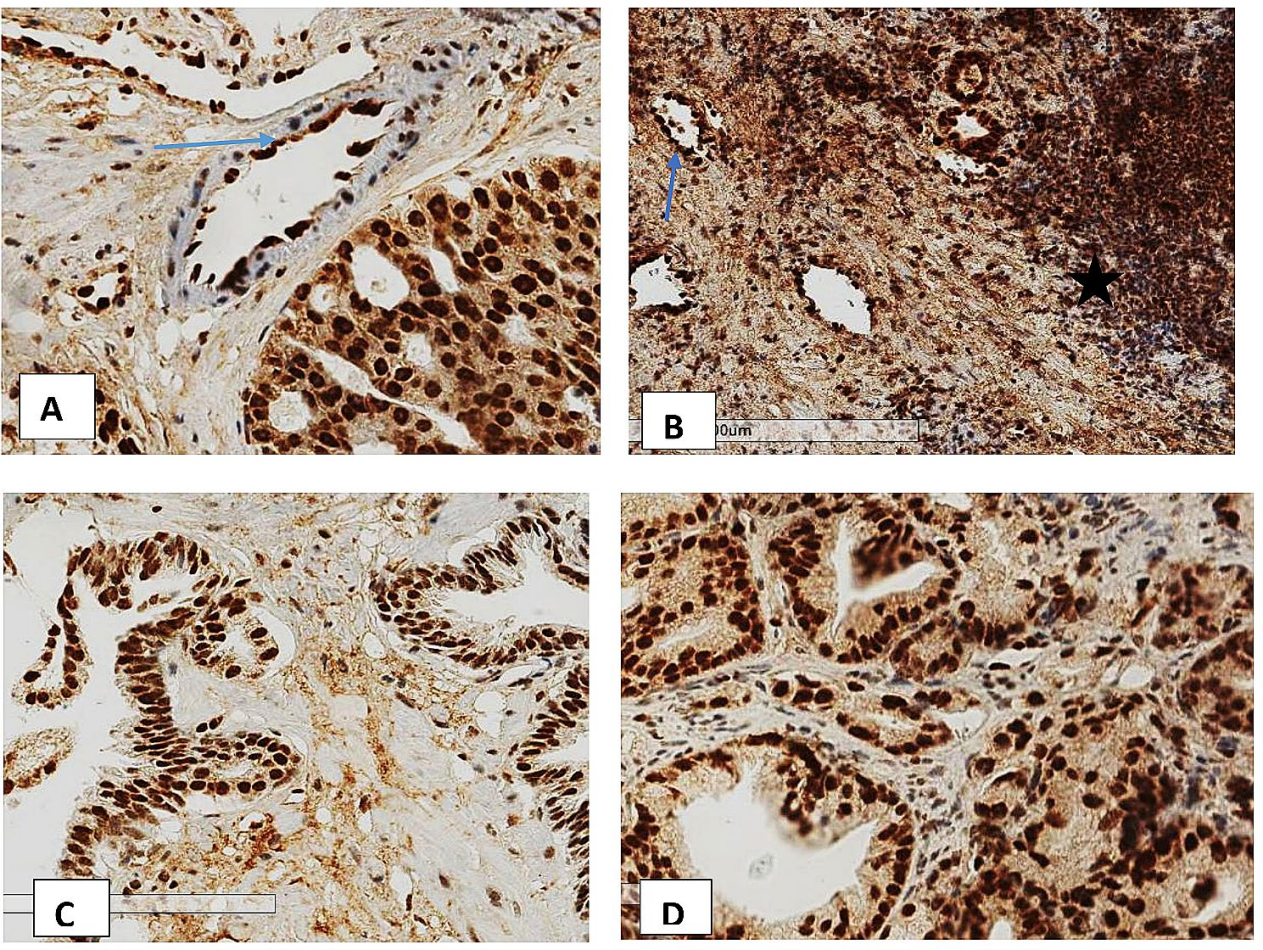
Shows ERG staining in different cells and premalignant glands. **A:** Strong ERG staining in the endothelial cells (arrow) and in cancer cells. **B:** ERG overstaining in lymphocytes (star) and endothelial cells (arrow). **C** and **D:** Show ERG staining in high-grade intraepithelial neoplastic glands adjacent to invasive cancer (left lower corner in panel **D**)

ERG was expressed in 75.4% (98/130) of the biopsies (see Fig. 3). Of the 98 ERG-positive biopsies, 32.7% (32/98) stained strongly, 52.0% (51/98) stained moderately, and 15.3% (15/98) stained weakly positive (see Fig. 1). Approximately 36.2% of the biopsies showed heterogeneous staining of the cells of interest. Staining was also observed in the high-grade lesions adjacent to the invasive carcinomas in a significant proportion of the biopsies. Lymphocytes, endothelial cells, and benign glands consistently provided internal controls (see Fig. 4).

### Association between ERG expression and Clinicopathological features of prostate cancer

At bivariable analysis, the odds of expression of ERG were 9.75 in tumors with a PSA of 19.1 ng/ml and above compared to tumors with a total PSA less than 9 though not statistically significant. The confidence interval (0.83-114.12) was quite wide and the *p*-value was just marginal (*p* = 0.07). For the PSA category of 9.1–19.0, the numbers were too few for meaningful comparison.

The odds of expression of ERG were 8.90 in grade group 3 compared to grade group 1. This is however not statistically significant due to a wide confidence interval (1.04–76.04) despite having a borderline *p*-value (0.05).

Although the odds of expression of ERG increased with increasing age groups, there was a significant statistical difference across the three age groups (see Table 2).

**Table 2:** Bivariable and multivariable logistic regression for Clinicopathological features and ERG protein expression

ERG status	Bivariable logistic regression			Multivariable logistic regression		
	OR	*P*-value	95% CI	OR	*P*-value	95% CI
Grade Group						
**1**	**Ref**			**Ref**		
2	1.78	0.36	0.52–6.13	1.73	0.569	0.26–11.51
3	8.90	0.05	1.04–76.04	2.52	0.440	0.24–26.18
4	1.78	0.36	0.52–6.13	2.89	0.369	0.29–29.10
5	1.36	0.56	0.48–3.82	0.66	0.557	0.17–2.61
Total PSA						
**0–9.0**	**Ref**			**Ref**		
9.1–19.0	1			1		
19.1 and above	9.75	0.07	0.83-114.12	8.74	0.109	0.62-123.77
Age groups						
**50–59**	**Ref**		**Ref**	**Ref**		**Ref**
60–69	1.5	0.63	0.28–7.91	1.08	0.952	0.084–13.99
> 70	3	0.17	0.62–14.62	1.86	0.622	0.16–22.06

In multivariable analysis, similar results were obtained as those in bivariable analysis. Although the odds of expression of ERG increase with the increase in the grade group/Gleason score from 1 to 4, the increase is not statistically significant as backed by the *p*-values > 0.05. Grade group 5 shows reduced odds (0.66) of expression of the protein but still it is not a statistically significant reduction. Similar findings are seen with serum PSA and age group. As shown in Table 2, there is no association between serum PSA, Gleason score, age group, and ERG expression.

## Discussion

We determined the prevalence of the prevalence of the ERG expression and its association with patient age, preoperative serum PSA, and Gleason score. In this study, the mean age of sample source patients at diagnosis was 74.6 ± 10.19 and the age range was from 50 to 106 years. The observed mean is comparable to the mean/median obtained in several studies conducted among Africans. A recent study conducted at the Uganda Cancer Institute (UCI) in Kampala reported a median age of 70 years. Similarly, studies from Nigeria and South Africa have reported mean ages of 70 years and 71 years for prostate cancer respectively [2, 20, 21]. Our mean age is also comparable to the mean observed in a study done in Jordan that reported a mean age of 77.4 years [22]. All the findings affirm that prostate cancer is a disease of men above 50 years of age as already known [23]. Preoperative serum PSA level was done for 79.2% of our patients. The mean and /or median PSA could not be calculated because it was observed that most patients that had PSA indicated as (> 100ng/ml) but not the absolute values. Absolute values were only provided for those that had PSA readings of less than 100ng/ml. In our study, grade group 5 was the commonest (27.69%), followed by grade group 1 (24.62%), grade groups 2 and 4 both had (16.92%) and the least was grade group 3 (13.85%). These results were similar to a study done in Nigeria where the author reports grade group 5 as the commonest (30.3%), followed by grade groups 4, 1, 3, and 2 [24]. Generally, 58.46% (76/130) of specimens had grade group 3 or more (high grade). The findings are in agreement with the observation that Africans generally present with advanced disease at diagnosis as also observed by Blackburn et al., (2019) and Okuku, Orem et al. (2016).

The TMPRSS2-ERG fusion gene is the most common genetic alteration seen in prostate cancer with very wide variations across different races, laboratories, cohorts, and zonal origin of the tissue used. Several other genes have also been reported to fuse with ERG in up to 10% of cases with ERG overexpression [11]. ERG protein is a known surrogate for fusion and its prevalence ranges from as low as 8%, to as high as 83% [12]. It is believed to be highest among Caucasians (50%), followed by African Americans (31.3%), and lowest among Japanese (15.9%) patients [25]. In our study, the fusion gene was detected in 75.4% of the biopsies as determined by the IHC expression of ERG, its surrogate marker [7]. Compared to the recent studies conducted in Ghana and South Africa that obtained a prevalence of 18% and 13% respectively, the prevalence of the fusion gene in our study is much higher [12, 13]. The differences could be explained by several factors, the most important among them being the genetic differences between West Africans, South Africans, and East Africans as well as differences in ERG detection methods. The South African study used real-time polymerase chain reaction (RT-PCR) which is a better detection method than IHC. In our setting, we had no access to RT-PCR nor could we access fluorescence insitu hybridization (FISH). Fortunately, IHC results are comparable to those obtained by RT-PCR, and FISH in a study done by Park et al., (2010) [26] No comparable studies were available from other East African countries for comparison.

Several studies have attempted to study the association between ERG expression and Gleason score/grade groups but the results are quite contradictory. Our study reveals that there is no difference in the expression of ERG protein across age groups, PSA levels, and grade groups in both bivariable and multivariable analyses. This is congruent with results obtained in studies done in several countries including China [16], USA [18, 27], and Korea [28]. Contrary to the above, several other studies have suggested that the fusion gene is common among men younger than 65 years, and those with low Gleason scores of 7(3 + 4) and 6 [12, 13, 29]. However, there are also a series of studies that have revealed that gene fusion is associated with a high Gleason score, high PSA, and aggressive disease [14, 30]. These contradictions can be attributed to the racial and genetic differences in the molecular pathogenesis of prostate cancer among the different races with Africans more likely to have TMPRSS2-ERG gene fusion through deletion [9, 12, 31]. Deletion type of fusion is associated with a high likelihood of metastasis in people of different racial backgrounds [16, 32]. Although immunohistochemical detection of ERG expression has a high sensitivity (89.6-96%) [33, 34] with comparable results to FISH and PCR, it cannot inform us whether the overexpression is due to fusion with TMPRSS2 or other genes as indicated earlier. Although the results show a high prevalence of ERG expression among patients in Southwestern Uganda, they are not entirely conclusive. Larger multicentre studies using better TMPRSS2-ERG detection methods (FISH/PCR) are needed to establish the definitive frequency of the ERG expression and the different gene fusions that determine its expression in our population.

### Limitations

Our study was not without limitations. The study employed IHC to detect the ERG protein as a surrogate for the TMPRSS2-ERG fusion gene and therefore we could not account for the other genes that can fuse with the ERG gene. Being a single-site study, our results may not be easily generalized to the population in Uganda and Africa at large. There is therefore a need for larger multisite studies to further this particular gene mutation and its distribution. Secondly, our study relied on already archived formalin fixed paraffin embedded prostate biopsies. We therefore could not control for cold ischemia and duration of fixation which all impact the quality of IHC staining.

## Conclusion

Similar to studies done at UCI and in several other African countries, a high proportion of prostate cancer patients in Southwestern Uganda have high-grade (grade group 3–5) disease at diagnosis. Compared to similar studies done among Africans, there is a significantly higher prevalence of ERG expression in our study. We have also observed that the expression of the fusion gene is independent of preoperative PSA, patient age, and Gleason score.

### Recommendation

With prostate cancer now the leading cause of cancer-related death among men in Uganda, there is a need to further understand the role of the ERG gene and confirm its prevalence. With such high expression of ERG protein, studies with better detection techniques (PCR, or FISH) are advised to confirm the above findings and their implications for medical practice concerning prostate cancer diagnosis.

## Data Availability

All the data that supports the findings of this study are available and archived in the Department of Pathology of Mbarara University of Science and Technology. All the data can be accessed on request after approval from the relevant bodies.
